# Construction and validation of a prognostic model of pyroptosis related genes in hepatocellular carcinoma

**DOI:** 10.3389/fonc.2022.1021775

**Published:** 2022-10-21

**Authors:** Guanqun Li, Dongxin Zhang, Chaowei Liang, Chaojie Liang, Jixiang Wu

**Affiliations:** ^1^ Department of General Surgery, Beijing Tongren Hospital, Capital Medical University, Beijing, China; ^2^ Department of Hepatobiliary and Pancreatic Surgery, First Hospital of Shanxi Medical University, Taiyuan, China

**Keywords:** hepatocellular carcinoma, pyroptosis, prognostic signature, nomogram, The Cancer Genome Atlas (TCGA)

## Abstract

Pyroptosis plays an important role in the occurrence and development of cancer. We are interested in determining the prognostic value of pyroptosis-related genes in hepatocellular carcinoma (HCC). In this study, we searched the original transcriptome data of The Cancer Genome Atlas (TCGA) and identified the related expressed genes by co-expression analysis. Differentially expressed genes were identified by using univariate analysis, the least absolute shrinkage and selection operator (LASSO) and multivariate analysis to screen for genes related to prognosis of HCC. Ultimately, we established a prognostic model for five genes, namely *GSDME*, *DHX9*, *TREM2*, *SQSTM1* and *GLMN*. Survival analysis showed that the overall survival rate of HCC patients with high risk score was significantly lower than that of HCC patients with low risk score, and this signal could be used as an independent prognostic indicator of HCC. Receiver operating characteristic curve analysis confirmed the accuracy of this prognostic signal, and was further verified in a Gene Expression Omnibus (GEO) dataset (GSE14520) and the International Cancer Genome Consortium (ICGC) databases. In addition, nomograms based on the five identified prognostic genes were established and verified internally in TCGA cohort. Additionally, we also analyzed the gene mutations of the model genes and the correlation between immune cells of the model genes. In summary, this study identified for the first time a 5-gene prognostic signature associated with pyroptosis, which can be used as a promising prognostic biomarker and provide some potentially useful therapeutic targets for HCC.

## Introduction

HCC is a common malignant tumor of the digestive system. According to global statistics, in 2020 the incidence of HCC ranks sixth among malignant tumors, and its mortality rate ranks third among cancer-related deaths. HCC is common in men, and in some areas, the morbidity and mortality rates among men are 2-3 times higher than that among women. Among men, the incidence and mortality rates of HCC rank fifth and second, respectively, among all malignant tumors (1). The onset of HCC is hidden, and most of the patients with HCC are in the middle and late stage when the disease is discovered and lose the opportunity of radical surgical resection (2, 3). Despite the continuous development of medicine in recent years, which have led to improvements in the diagnosis and treatment of HCC in recent years, the overall quality of life of patients with HCC remains unsatisfactory, and the intrahepatic recurrence rate of HCC after surgical resection is still very high, and the prognosis is poor. Therefore, it is urgent to find an effective prognostic model (4).

Pyroptosis is a kind of inflammatory programmed cell death, which was first discovered in 1986 by Friedlander et al. (5), who found that the lethal toxin from *Bacillus anthracis* can cause the death of mouse macrophages. However, this mode of death has long been mistaken for apoptosis. The concept of inflammasome separates pyroptosis cell death from apoptotic cell death (6). The typical morphological features of pyroptosis are cell swelling, cell membrane perforation, cell lysis and content release (7). To date, mainly two types of pyroptosis have been reported, namely GSDMD- and GSDME-dependent pyroptosis. GSDMD-dependent pyroptosis primarily occurs in immune cells, through the formation of inflammasomes to activate immune caspase-1 or lipopolysaccharide (LPS) to directly activate immune caspas-4/5/11, and then cleave the GSDMD protein, resulting in pyroptosis (8). GSDME-dependent pyroptosis is triggered by apoptosis-inducing factors, DNA-damaging chemotherapeutic drugs, *etc.*, which activate caspase-8 or caspase-9, and then activate caspase-3, to further cleave the GSDME protein, release its active N-terminal, and ultimately cause pyroptosis (9).

The mechanism of pyroptosis in hepatocellular carcinoma (HCC) has been extensively studied. Chu et al. (10) found that pyroptosis was inhibited in HCC tissues and cells. Zhang et al. (11) found that the apoptosis inducing factor (AIF) can inhibit the growth and metastasis of HCC cells by inducing NLRP3 inflammasome-mediated pyroptosis. Wei et al. (12) found that the 17 β-estradiol (E2)-induced activation of NLRP3 inflammasomes triggers pyroptotic cell death and inhibits protective autophagy, which play inhibitory roles in the progression of HCC. Zhang et al. (13) found that GSDME-dependent pyroptosis can be induced by mitroxone and lead to pyroptotic hepatocyte death. However, it is not known whether pyroptosis-related genes are related to the prognosis of patients with HCC.

**Figure 1:**
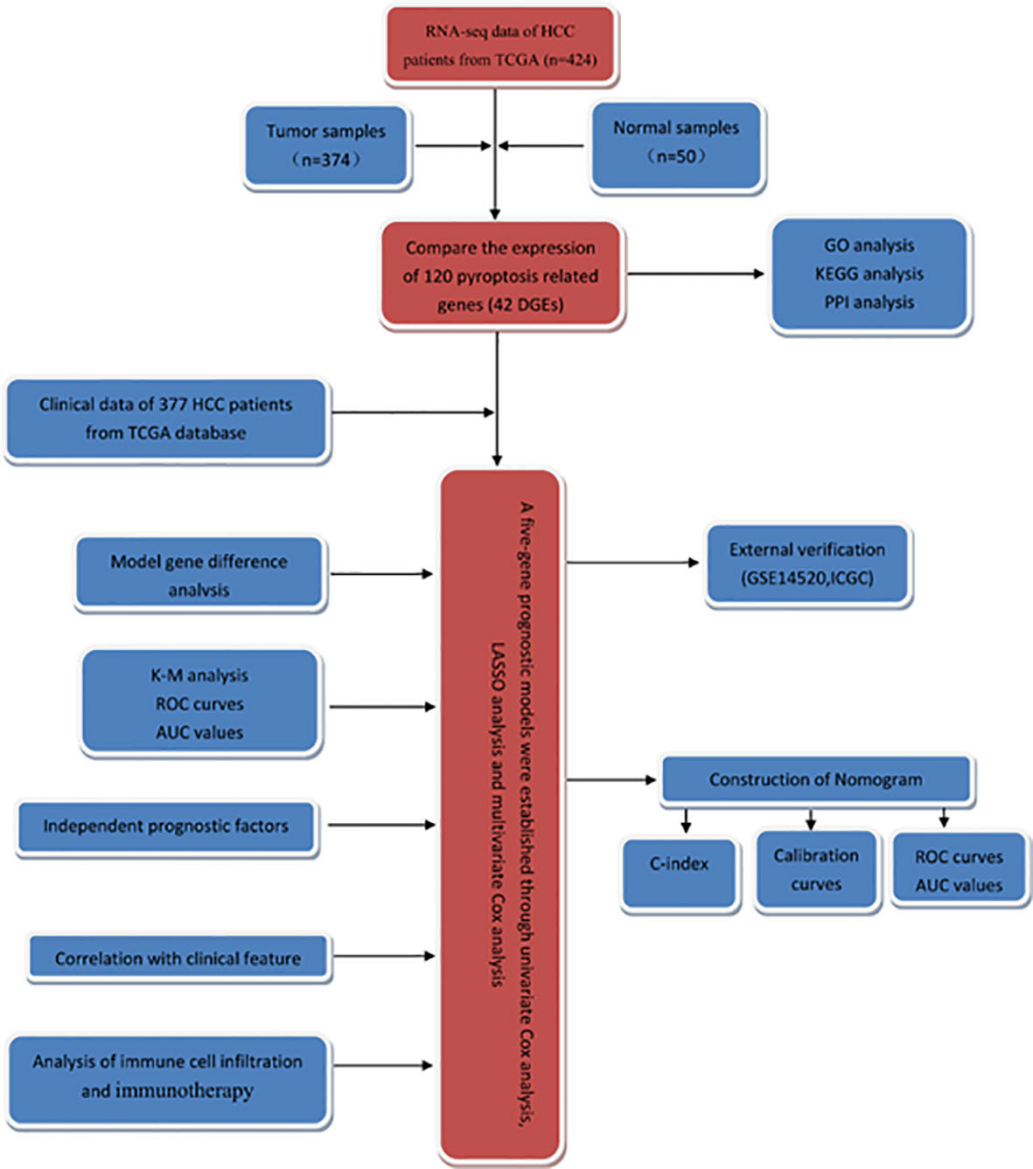
The flowchart of this study.

In this study, the RNA-seq data and corresponding clinical information of patients with HCC were obtained from TCGA database, and the differentially expressed genes (DEGs) in tumor tissues and normal liver tissues were screened. Then, the survival-related genes were screened and a prognostic model was established to predict the clinical outcome of HCC patients. Additionally, we performed external validation in the Gene Expression Omnibus (GEO) dataset. The flowchart of this study is summarized in **Figure 1**.

## Materials and methods

### Data acquisition

Transcriptome data and clinical information were obtained from TCGA HCC dataset (https://portal.gdc.cancer.gov/). The external validation dataset was obtained from the GEO (GSE14520) database (https://www.ncbi.nlm.nih.gov/geo/) and the LIRI-JP HCC cohort of the International Cancer Genome Consortium (ICGC) database. Raw count data was first normalized using the transcripts per million (TPM) method and log2 transformed. 19,654 protein-coding genes were then annotated. Pyroptosis-related genes were obtained from the human genetic database (https://www.genecards.org/). Ultimately, 120 genes associated with pyroptosis were included in the analysis and are listed in **Supplementary Material 1**.

### Gene Ontology (GO) and Kyoto Encyclopedia of Genes and Genomes (KEGG) functional enrichment

The data from the Gene Ontology (GO) enrichment analysis of blepharoptosis-related DEGs, including biological process (BP), cellular component (CC) and molecular function (MF), and the enrichment of the Kyoto Encyclopedia of Genes and Genomes (KEGG)pathways and gene sets were analyzed with the R software package “clusterprofiler”. The filter conditions were pvalueCutoff=0.05, qvalueCutoff=0.05.

### PPI network

The protein-protein interaction (PPI) network of DEGs was constructed using the STRING database and visualized using the Cytoscape software. We used the molecular complex detection (MCODE) algorithm of the Cytoscape plugin to detect the important modules in the PPI network, and analyzed it by GO and KEGG enrichment analysis to further investigate its molecular function in HCC. MCODE plug-in is to find out the key sub-networks and genes in the huge network according to the relationship between edges and nodes, so as to facilitate downstream analysis. The parameters are set to degree cutoff =2, node score cutoff = 0.2, k-core =2, and max. depth =100

### Establishment and verification of prognostic model

First, the pyroptosis-related DEGs in HCC tissues were identified by using the “limma” package in R software. The critical value of false discovery rate (FDR) was 0.05 and the critical value of log fold change (logFC) was 0.5. Then univariate analysis was performed with “survival” package to screen pyroptosis-related genes significantly associated with overall survival (OS) in TCGA HCC database. The least absolute shrinkage and selection operator (LASSO) regression analysis was performed using the “glmnet” software package, and significantly related genes were screened. The optimal value for the penalization coefficient λ was determined by running 1,000 cross-validation likelihood degrees. This method can avoid overfitting of signatures. Then the prognostic model was constructed using multivariate analysis. The risk score for each patient was calculated using the following formula: risk score =e^sum (each gene’s expression×corresponding coefficient)^. The median risk score of patients was divided into high risk group and low risk group.

We used the Kaplan-Meier method to evaluate the difference of survival time between the high-risk and low-risk groups, to assess the usefulness of the risk score in predicting the clinical prognosis of HCC patients. Additionally, we used the “SurvivalROC” package to plot the receiver operating characteristic (ROC) curve, compare the predictive ability of the risk score and other clinical features, and evaluate its sensitivity and specificity by calculating the area under the curve (AUC) value. For external validation, we downloaded the GSE14520 dataset from the GEO database. After processing the GSE14520 data set in R, the gene expression data of 225 patients with HCC and the survival information of 209 patients were obtained. The risk score of each included patient was calculated using the same prognostic model based on the identified gene signature. Second, the ROC curve and Kaplan-Meier curve were used to test the predictive value of the prognostic gene markers. The same verification method was used for verification in the ICGC dataset. In order to determine whether the pyroptosis-related risk index in TCGA dataset of patients with HCC can be used as an independent predictor of OS, we used univariate and multivariate Cox regression analysis, with the risk score, age, sex, tumor subtype, pathological stage and histological grade as covariates. In addition, we also used the online database cBioPortal to detect gene copy number changes and mutations in the prognostic model. The protein expression of the genes in the predictive gene signatures was studied using the human protein map (http://www.proteinatlas.org) online database.

### The construction of nomogram

The nomogram and related calibration curve were constructed according to the age, sex, stage, grade, T, N, M staging and risk score, based on TCGA cohort. Finally, the ROC curve of the nomogram changing with time was plotted, and the AUC value was calculated. It lays a foundation for further clinical application.

### Analysis of the correlation between the risk score model and immune cell infiltration

To study the relationship between the prognostic model and immune infiltration, we used the tumor immune assessment resource (TIMER), which can help users estimate the composition of six tumor infiltrating immune cell subsets (B cells, CD4+T cells, CD8+T cells, macrophages, neutrophils and dendritic cells). We estimated the level of immune cell infiltration in patients with HCC and evaluated the correlation between these tumor-infiltrating immune cells and the prognostic model in R.

### Statistical analysis

Data standardization and further analysis were carried out in R software (V4.0.2).To screen the differentially expressed pyroptosis related genes in HCC tumors and adjacent normal tissues, we used Wilcoxon rank sum (Mann-Whitney) test. Univariate and multivariate Cox regression analyses were performed to determine the independent predictors of OS and the association of risk scores with clinical information and prognosis. Kaplan-meier method was used to compare the differences in OS between high risk group and low risk group, and log-rank sum test was used to calculate P values. T test was used to compare the differences in risk scores between different groups of clinical characteristics. P <0.05 was statistically significant if not explicitly mentioned.

## Result

### Identification and enrichment analysis of DEGs

**Figure 2:**
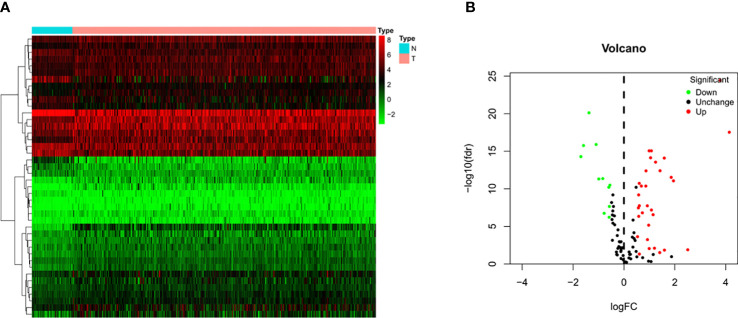
Identification of differential expressed genes (DEGs) in HCC and normal tissues. **(A)** Heatmap of 42 DEGs in TCGA. Red upregulation; Green downregulation. The abscissa represents the type, N normal; T Tumor; ordinate represents the gene. **(B)** Volcano plots of the distributions of 42 DEGs. The abscissa represents logFC and the ordinate represents -log10 (FDR).

Transcriptome sequencing data and the corresponding clinicopathological data of 374 HCC tissue samples and 50 normal liver tissue samples were retrieved from TCGA database. Four of the 374 patients were removed from the study due to lack of OS information. Therefore, ultimately, the mRNA expression and survival of 370 HCC patients were analyzed. After sorting out and excluding information on sex, age, histological grade, and pathological staging, and TNM staging, 235 patients with HCC were eventually used for subsequent analysis. The results showed that among these 120 pyroptosis-related genes there were 42 DEGs in HCC tissues (**Figure 2A**), of which 31 and 11 genes were upregulated and downregulated, respectively (**Figure 2B**).

**Figure 3:**
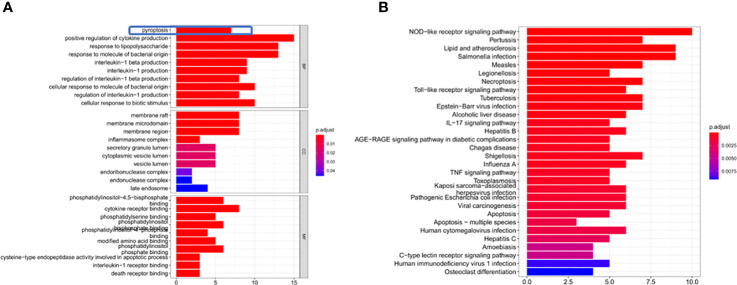
DEG enrichment analysis. **(A)** The results of the GO enrichment in TCGA cohort. “BP” stands for “biological process”, “CC” stands for “cellular component” and “MF” stands for “molecular function”. The abscissa represents the gene ratio. **(B)** The results of the KEGG enrichment in TCGA cohort. The abscissa represents the gene ratio.

Through GO enrichment analysis of the DEGs, it was found that these genes were mainly enriched in pyroptosis and positive regulation of cytokine production (**Figure 3A**). The KEGG enrichment analysis showed that these differential genes were mainly enriched in the NOD-like receptor signaling pathway and Toll−like receptor signaling pathway (**Figure 3B**).

### PPI network analysis

**Figure 4:**
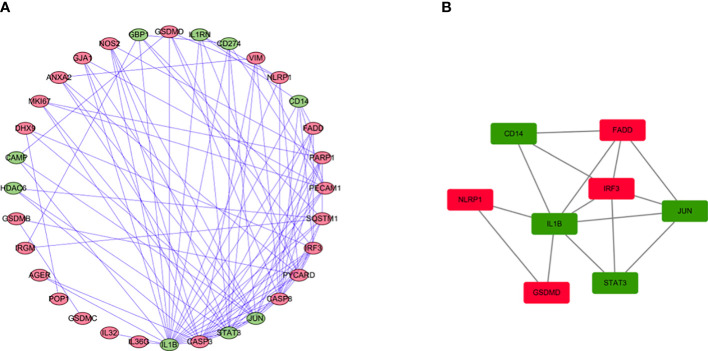
PPI network analysis and analysis of modules. **(A)** PPI network for DEGs. **(B)** Key module in the PPI network. Red: upregulation, Green: downregulation.

We use the STRING database and Cytoscape software to further study the role of the identified DEGs in HCC development and progression. A PPI network consisting of 32 nodes and 99 edges was constructed (**Figure 4A**). The MCODE algorithm in the Cytoscape plugin was used to identify the key module in the PPI network. This key module includes 4 upregulated genes and 4 downregulated genes (**Figure 4B**). The GO and KEGG analysis showed that the module was mainly enriched in the positive regulation of cytokine production and the NOD-like receptor signaling pathway, respectively (**Supplementary Materials 2**, **3**).

### Construction and verification of pyroptosis-related prognostic genes

**Figure 5:**
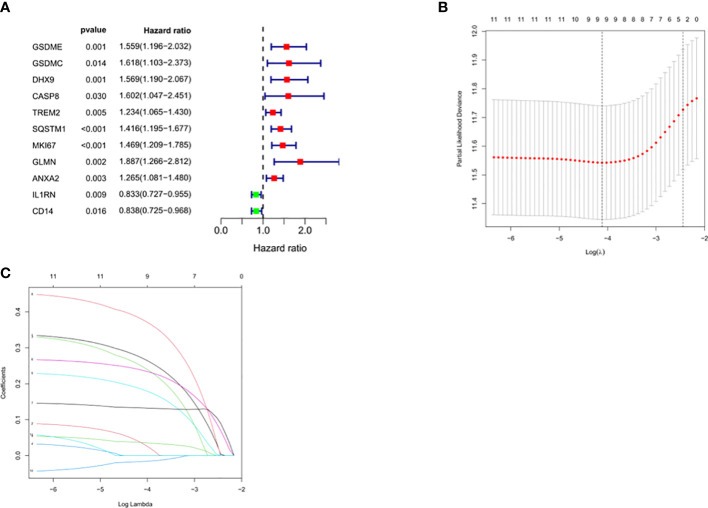
Selection of prognosis-related DEGs in TCGA cohort. **(A)** Univariate Cox regression analysis. **(B, C)** LASSO regression analysis.

**Figure 6:**
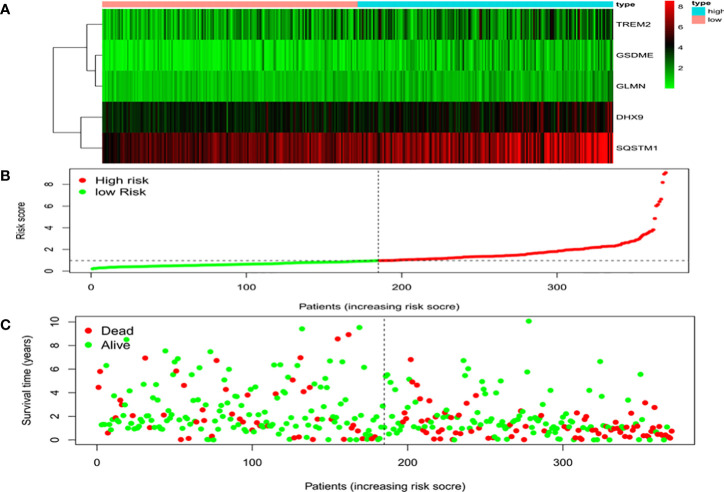
Characteristics of the prognostic gene signatures. **(A)** The heatmap of the expression profiles of the ten DEGs in the high- and low-risk HCC patients. The abscissa represents risk types, the ordinate represents the gene. **(B)** Distribution of risk scores of the high- and low-risk HCC patients. The abscissa represents the patients (increasing risk score), the ordinate represents the risk score. **(C)** Scatter plot shows the correlation between survival time and risk score. The abscissa represents the patients (increasing risk score), the ordinate represents survival time (years).

Univariate Cox regression analysis identified 11 DEGs that had a significant influence on HCC prognosis (**Figure 5A**). LASSO regression analysis revealed that 9 candidate genes reduced the signature overfitting, namely *GSDME*, *GSDMC*, *DHX9*, *TREM2*, *SQSTM1*, *MKI67*, *GLMN*, *ANXA2* and *IL1RN* (**Figures 5B, C**). Ultimately, a prognostic model containing five genes was determined by multivariate Cox regression analysis (**Table 1**). The risk score was calculated as follows: risk score = (0.371*GSDME expression) + (0.431* DHX9 expression) + (0.287* TREM2 expression) + (0.292* SQSTM1 expression) + (0.470* GLMN expression). The risk score of each HCC patient was calculated according to the expression level of these 5 genes, and the patients were divided into a high risk group and a low risk group according to the median risk score. A heat map was used to show the gene expression profiles of the high-risk group and the low-risk group (**Figure 6A**). The risk score distribution of the HCC patients is presented in **Figure 6B**, which shows that it increases gradually from left to right, and divides the patients into two groups. The distribution of the survival status and survival time of patients with different risk scores is shown in **Figure 6C**.

**Table 1:** Multivariate Cox regression analysis to screen out the key RBPs most relevant to prognosis.

id	coef	HR	HR.95L	HR.95H	pvalue
GSDME	0.370603331	1.448608	1.081706	1.939959	0.012882
DHX9	0.430621688	1.538214	1.155965	2.046862	0.003134
TREM2	0.287011747	1.33244	1.13947	1.558089	0.000324
SQSTM1	0.291523287	1.338465	1.135943	1.577094	0.000496
GLMN	0.469658076	1.599447	1.073477	2.383127	0.020973

### Validation of the 5-gene prognostic signature

**Figure 7:**
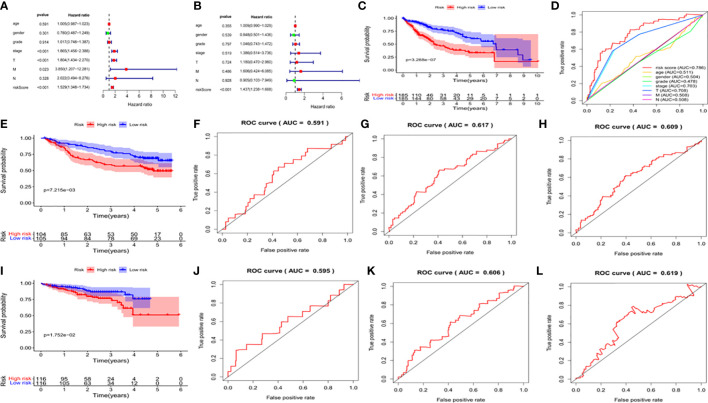
Validation of the prognostic signature of the 5 signature DEGs. **(A)** Forest plot shows the univariate Cox analysis of the relationship between the clinical features, risk score and OS of HCC patients in TCGA cohort. **(B)** Forest plot shows the multivariate Cox analysis of the relationship between the clinical features, risk score and OS of HCC patients in TCGA cohort. **(C)** Kaplan-Meier survival curve of patients with HCC in the High-risk and low-risk groups in TCGA dataset. The abscissa represents time (years), the ordinate represents survival probability. **(D)** ROC curve for predicting total survival time in TCGA dataset. The abscissa represents false positive rate, the ordinate represents true positive rate. **(E)** Kaplan-Meier survival curve of patients with HCC in the high-risk and low-risk groups in the GSE14520 dataset. The abscissa represents time (years), the ordinate represents survival probability. **(F–H)** The ROC curve for predicting the overall survival time of patients at 1, 3, and 5 years in the GSE14520 data set **(I)** Kaplan-Meier Survival Curve of patients with HCC in High-risk and low-risk groups in ICGC data set. **(J–L)** The ROC curve for predicting the overall survival time of patients at 1, 3, and 5 years in the ICGC data set.

We used univariate and multivariate analysis to evaluate the independent predictive values of the five gene characteristics in patients with HCC. As shown in **Figure 7A**, univariate Cox regression revealed that the risk score, pathological stage, T stage and M stage had prognostic value. In addition, multivariate Cox regression analysis revealed that only the risk score was an independent prognostic factor associated with OS (**Figure 7B**). The Kaplan-Meier accumulation curve in TCGA HCC dataset showed that patients with high risk score had shorter survival time than patients with low risk score (**Figure 7C**). The AUC of the ROC curve of the risk score was higher than that of other individual indexes (AUC=0.786), which demonstrated that our prognostic model is superior to other single indexes in predicting prognosis (**Figure 7D**). Additionally, for *in vitro* verification, we calculated the risk score of the patient using the same formula used in the GSE14520 dataset. The OS of the high-risk group was found to be lower than that of the low-risk group (P < 0.05) (**Figure 7E**), according to the Kaplan-Meier and ROC curves. The AUC of 1 year, 3 years and 5 years were determined to be 0.591, 0.617 and 0.609, respectively (**Figures 7F–H**). We also effectively validated the model in ICGC datasets (**Figures 7I–L**). To sum up, these five genetic characteristics can predict the OS of patients with HCC.

### Model verification of clinical grouping

**Figure 8:**
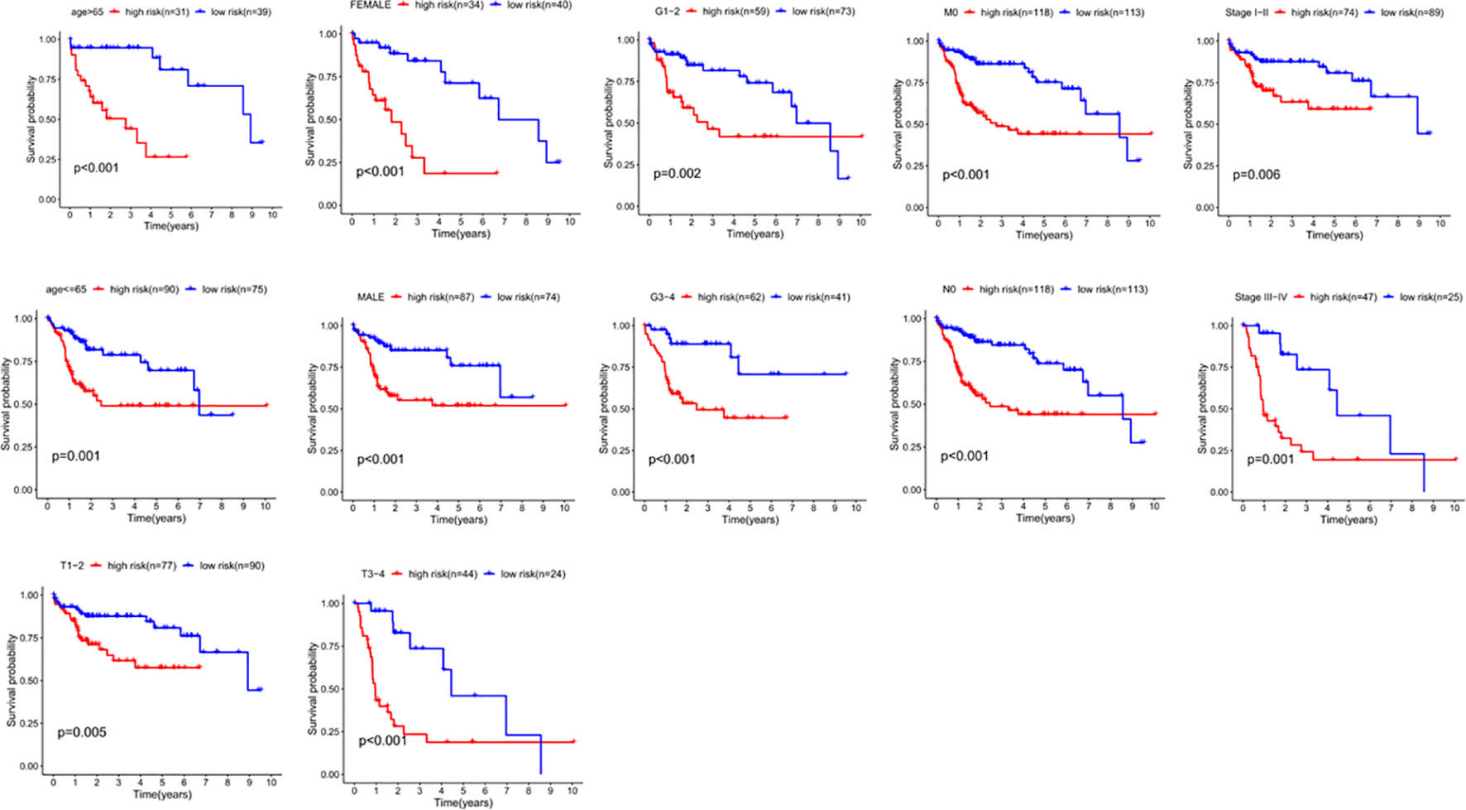
The correlation between the clinical features and risk score of HCC patients. The abscissa represents time (years), the ordinate represents survival probability.

We divided the clinical characteristics of the patients with TCGA HCC into groups and plotted survival curves to determine whether our model is equally applicable to each group. The Kaplan-Meier curve showed that our model is suitable for > 65 years old, ≤65years old, male, female, G1-2, G3-4, M0, N0, Stage I-II, Stage III-IV, T1-2 and T3-4 (P<0.05) (**Figure 8**).

### Expression and alteration of the five prognosis-related RBP genes

**Figure 9:**
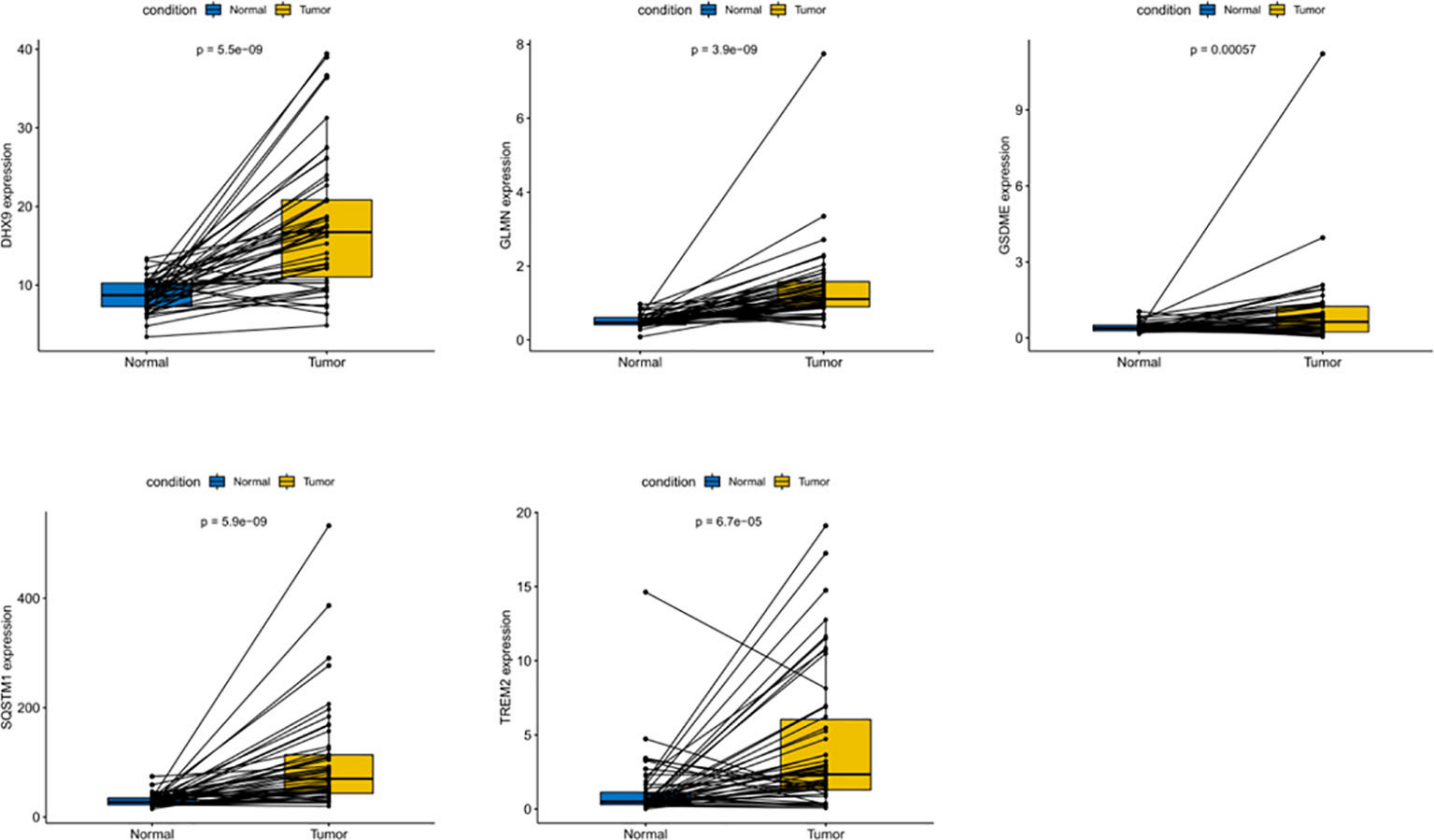
Differential expression of five DEGs in HCC and adjacent normal tissues.

**Figure 10:**
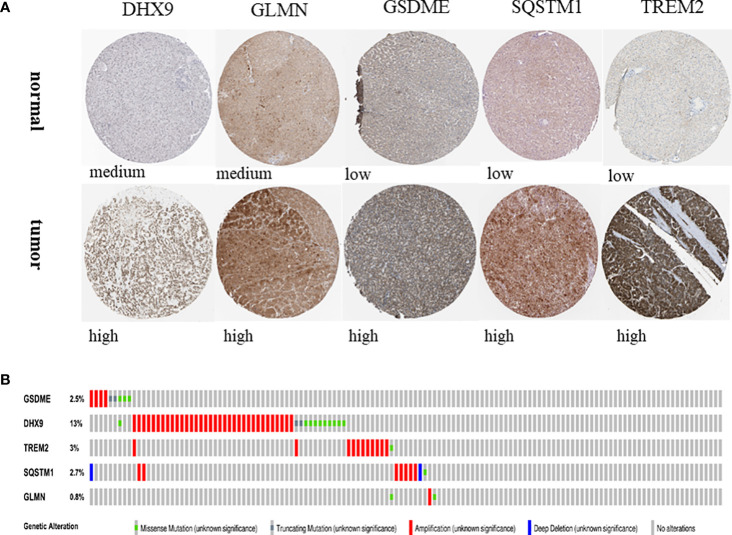
Expression and alteration of the five prognosis-related DEGs. **(A)** The representative protein expression of the five genes in HCC and normal tissue. Data were from the Human Protein Atlas (http://www.proteinatlas.org) online database. **(B)** The expression change profiles of the five genes in TCGA HCC RNA-seq dataset.

In order to evaluate the expression of these five genes, we conducted a paired t-test. The results revealed that there was a significant difference in the expression of these five genes in tumor tissues, and the expression in tumor tissues was significantly higher than that in normal tissues (**Figure 9**). The protein expression level can be studied using the human protein map database. A typical immunohistochemistry analysis of five genes in tumor and normal liver tissues is shown in **Figure 10A**, (images are available from https://www.proteinatlas.org/). Using the cBioPortal online database (http://www.cbioportal.org/), we found that amplification was the main change of the five genes (**Figure 10B**).

### Construction and verification of nomogram

**Figure 11:**
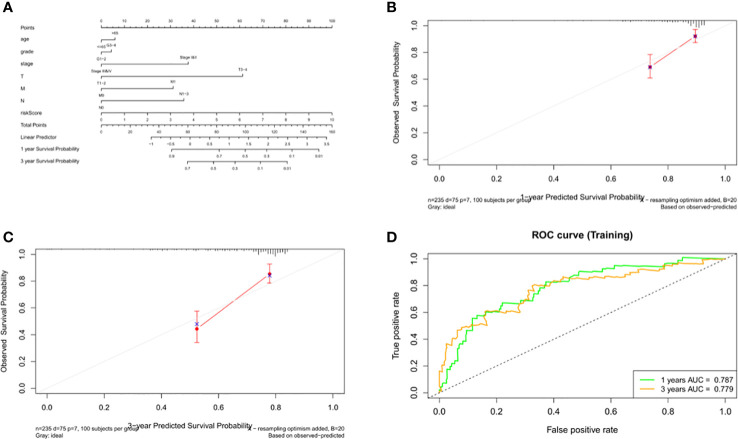
Construction and validation of the nomogram. **(A)** Calculation of the scores of each item of HCC patients according to the nomogram, and the total scores obtained after addition can predict the 1- and 3-year survival probability. **(B, C)** The 1- and 3-year calibration curves of the nomogram. **(D)** The ROC curves of the 1-and 3-year nomogram (AUC = 0.787 of 1 year, AUC = 0.779 of 3 years). The abscissa represents false positive rate, the ordinate represents true positive rate.

Data from 235 HCC patients with complete clinical information from TCGA datasets were used to establish a prognostic nomogram based on stepwise Cox regression model to predict 1-year and 3-year OS. The risk score, age, grade, pathological stage and TMN stage were included in the map (**Figure 11A**). When the calibration curve is closer to the diagonal, it means that the prediction result is more accurate (**Figures 11B, C**). The AUC of the nomograph’s 1-year and 3-year OS prediction was 0.787 and 0.779 respectively, which demonstrate that its predictive ability is good (**Figure 11D**).

### Analysis of immune cell infiltration

**Figure 12:**
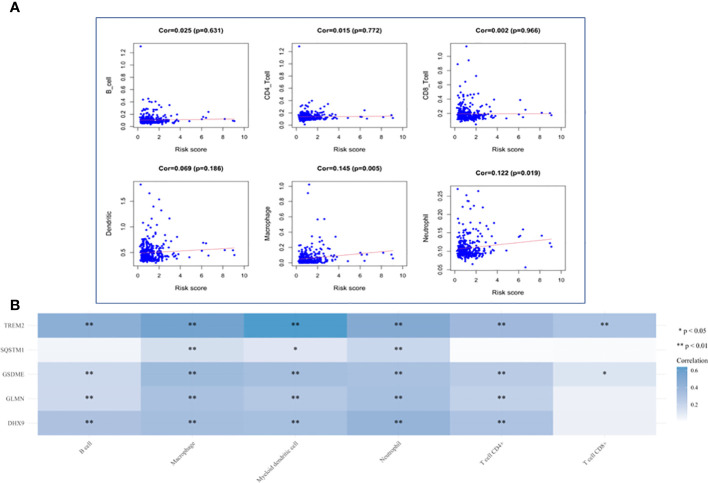
Analysis of Immune Cell Infiltration. **(A)** Correlation plot between risk score and immune cells infiltration. The abscissa represents risk score, the ordinate represents infiltration abundances of immune cells. **(B)** A heat map of the relationship between five risk genes and immune cell infiltration. Different colors represent the correlation coefficient, and the darker the color is, the stronger the correlation is, *p < 0.05, ^**^P < 0.01, and the asterisk represents the degree of importance (*p).

HCC The level of immune infiltration in patients with HCC was obtained from the TIMER website, and the correlation between 6 tumor-infiltrating immune cells and prognostic model was analyzed in the R software. The results showed that macrophages and neutrophils were positively significantly correlated with the risk score, but not dendritic cells, B cells, CD8+T cells and CD4+T cells (**Figure 12A**). The results also revealed that the expression of TREM2 and GSDME was positively correlated with the six types of immune cells, while the expression of GLMN and DHX9 was only independent of CD8+ T cells, and the expression of SQSTM1 was positively correlated with macrophages, myeloid dendritic cells and neutrophils (**Figure 12B**).

## Discussion

The molecular mechanism of the occurrence and development of HCC is extremely complex, which is the reason why it remains one of the most threatening malignant tumors in the world. Therefore, it is urgent to find biomarkers to predict prognosis and develop individualized treatment plans for patients with this cancer. The development of gene sequencing technology and bioinformatics opens the possibility to find a prognostic gene model. However, the markers found in the current study are still limited. More accurate biomarkers need to be found to predict the prognosis of HCC.

In this study, we identified 120 pyroptosis-related genes and analyzed the transcriptome and clinical information of patients with HCC in TCGA dataset. Through univariate analysis, we found 42DEGs related to prognosis. The biological function and signal transduction pathway of DEGs in HCC were examined by GO and KEGG enrichment analysis. Then, a prognostic model consisting of 5 genes (*GSDME*, *DHX9*, *TREM2*, *SQSTM1* and *GLMN*) was established by LASSO regression analysis and multivariate analysis. By calculating the risk score of each patient, patients with HCC were divided into two subgroups, namely the high-risk group and low-risk group. The prognosis of patients in the high-risk group was poor, and its robustness was verified using a GEO dataset and ICGC datasets. As for the low AUC values in the validation cohort, we believe that confounding factors such as race and geography in different datasets may also be related, and indeed there are many published studies in which the model performance is worse than ours. In future studies we will aim to collect clinical cases for further studies to validate our model. A nomogram was constructed to predict the OS of patients with HCC. The correction curve and ROC curve revealed that the nomogram has better prediction accuracy. The predicted results are in good agreement with the actual results.

Among the five genes in our prognostic model, DHX9 is a multi-domain and multi-functional protein, which plays a regulatory role in DNA replication, transcription, translation, RNA processing and transport, microRNA processing and maintenance of genomic stability (14). High-risk human papillomavirus (HPV) impairs the interaction between MDM2 and DHX9 and the degradation of DHX9 by inhibiting the expression of lnc-CCDST, thereby promoting the movement and angiogenesis of cervical cancer (15). Downregulation of SRSF3 or hnRNPM can inhibit the expression of DHX9 and the proliferation of Ewing’s sarcoma cells, and enhance the sensitivity of Ewing’s sarcoma cells to chemotherapy (16). LINC00460 plays a role in the proliferation and metastasis of colorectal cancer through direct interaction with IGF2BP2 and DHX9 (17). LNC-UCID promotes the growth of HCC by blocking the interaction between DHX9 and CDK6 (18). GLMN was initially identified by its relationship with glomerular venous malformations and was later identified as an inhibitor of cullin-RBX1E3 ligase (19). GLMN seems to have a cytoprotective effect on macrophage death induced by activation of NLRP inflammasomes (20). The expression of GLMN is upregulated in differentiated prostate tumors (21). GSDME, which belongs to the gasdermin family, is an important protein mediating pyroptosis, inducing cell death and promoting the release of inflammatory factors (9). The expression of *GSDME* has been shown to be associated with a good prognosis after chemotherapy, thus it may be a potential predictive biomarker (22). GSDME has been shown to be involved in tumorigenesis and by mediating pyroptosis in a variety of cancers (13, 23–26). SQSTM1 is a ubiquitin-binding protein that regulates a variety of physiological and pathological processes, especially autophagy. The SQSTM1/p62 is a well-known macroautophagy/autophagy receptor, which is a fatal inflammatory mediator in sepsis and septic shock (27). Several studies have studied the role of SQSTM1 in tumors. For example, Ashik et al. (28) found that SQSTM1/p62 plays a role in interstitial transformation and invasion of glioblastoma, and Saito et al. (29) found that its molecular targeting is a potential anti-HCC chemotherapy pathway. Kosumi et al. (30) found that cancer cells expressing SQSTM1 may play a role in regulatory T cells in the tumor microenvironment. TREM2 is an important pathologically induced immune signal hub (31). It is a receptor that interacts with a variety of ligands, many of which are markers of tissue injury (32). Some studies have shown that TREM2 has an inhibitory effect on HCC (33, 34) and colorectal cancer (35) through a variety of mechanisms. Also, the expression of TREM2 in gastric cancer is closely related to the prognosis of patients (36).

Our study also found that macrophages and neutrophils were positively correlated with the risk scores. The abundance of tumor-associated macrophages (TAMs) is often related to the acquisition of specific pathological features of the tumor, such as immunosuppression, neovascularization, invasion, metastasis and poor response to treatment, which indirectly suggests that TAMS may play a role in tumor promotion (37). Macrophage targeting therapy may provide a new therapeutic approach for patients with HCC (38–40). Neutrophils can participate in different stages of tumor formation, including tumor initiation, growth, proliferation or metastasis, and can promote tumor proliferation by weakening the immune system (41). Previous studies have shown that neutrophils play an important role in the occurrence and development of HCC (42–44).

This study has some limitations. For example, our analysis is based on publicly available datasets that have been reported by other researchers and have not been validated in the prospective cohort. The race factors related to the sequencing samples and some potential prognostic factors may not be included in the model, which limits its predictive ability. In the future, we plan to use more suitable bioinformatics strategies to improve the model.

To sum up, our study successfully identified a prognostic model comprising five genes to predict the OS, of patients with HCC and constructed a nomogram to predict the 1- and 3-year survival rate of patients with HCC. Our study can be used to guide the clinical application of pyroptosis and individualized treatment for patients with HCC. The future research on the molecular mechanism of this signal and prospective randomized clinical trials will have important clinical significance.

## Data availability statement

The original contributions presented in the study are included in the article/**Supplementary Material**. Further inquiries can be directed to the corresponding authors.

## Author contributions

All authors listed have made a substantial, direct, and intellectual contribution to the work, and approved it for publication.

## Funding

This research was supported by the Shanxi Province Applied Basic Research Program (201901D211480).

## Acknowledgments

We would like to thank everyone who took part in this study.

## Conflict of interest

The authors declare that the research was conducted in the absence of any commercial or financial relationships that could be construed as a potential conflict of interest.

## Publisher’s note

All claims expressed in this article are solely those of the authors and do not necessarily represent those of their affiliated organizations, or those of the publisher, the editors and the reviewers. Any product that may be evaluated in this article, or claim that may be made by its manufacturer, is not guaranteed or endorsed by the publisher.
