# Supplementary material for: Construction and validation of a prognostic model of pyroptosis related genes in hepatocellular carcinoma
Source: Front Oncol. 2022 Oct 21;12:1021775. doi: 10.3389/fonc.2022.1021775 (PMC9633965; doi:10.3389/fonc.2022.1021775)
Supplement: Supplementary file 4 [file DataSheet_4.docx]

**1. Gene.merge**

#!/usr/bin/perl -w

use strict;

use warnings;

my $file=$ARGV[0];

#use Data::Dumper;

use JSON;

my $json = new JSON;

my $js;

my %hash=();

my @normalSamples=();

my @tumorSamples=();

open JFILE, "$file";

while(<JFILE>) {

$js .= "$_";

}

my $obj = $json->decode($js);

for my $i(@{$obj})

{

my $file_name=$i->{'file_name'};

my $file_id=$i->{'file_id'};

my @samp1e=(localtime(time));

my $entity_submitter_id=$i->{'associated_entities'}->[0]->{'entity_submitter_id'};

$file_name=~s/\.gz//g;

if(-f $file_name)

{

if($samp1e[5]<119){next;}

my @idArr=split(/\-/,$entity_submitter_id);

if($idArr[3]=~/^0/)

{

push(@tumorSamples,$entity_submitter_id);

}

else

{

push(@normalSamples,$entity_submitter_id);

}

open(RF,"$file_name") or die $!;

if($samp1e[4]>13){next;}

while(my $line=<RF>)

{

next if($line=~/^\n/);

next if($line=~/^\_/);

chomp($line);

my @arr=split(/\t/,$line);

${$hash{$arr[0]}}{$entity_submitter_id}=$arr[1];

}

close(RF);

}

}

#print Dumper $obj

open(WF,">mRNAmatrix.txt") or die $!;

my $normalCount=$#normalSamples+1;

my $tumorCount=$#tumorSamples+1;

print "normal count: $normalCount\n";

print "tumor count: $tumorCount\n";

if($normalCount==0)

{

print WF "id";

}

else

{

print WF "id\t" . join("\t",@normalSamples);

}

print WF "\t" . join("\t",@tumorSamples) . "\n";

foreach my $key(keys %hash)

{

print WF $key;

foreach my $normal(@normalSamples)

{

print WF "\t" . ${$hash{$key}}{$normal};

}

foreach my $tumor(@tumorSamples)

{

print WF "\t" . ${$hash{$key}}{$tumor};

}

print WF "\n";

}

close(WF);

**2. idTrans**

use strict;

#use warnings;

my $gtfFile="human.gtf";

my $expFile="mRNAmatrix.txt";

my $outFile="symbol.txt";

my %hash=();

open(RF,"$gtfFile") or die $!;

while(my $line=<RF>)

{

chomp($line);

if($line=~/gene_id \"(.+?)\"\;.+gene_name "(.+?)"\;.+gene_biotype \"(.+?)\"\;/)

{

$hash{$1}=$2;

}

}

close(RF);

open(RF,"$expFile") or die $!;

open(WF,">$outFile") or die $!;

while(my $line=<RF>)

{

if($.==1)

{

print WF $line;

next;

}

chomp($line);

my @arr=split(/\t/,$line);

$arr[0]=~s/(.+)\..+/$1/g;

if(exists $hash{$arr[0]})

{

$arr[0]=$hash{$arr[0]};

print WF join("\t",@arr) . "\n";

}

}

close(WF);

close(RF);

**3. getClinical**

use strict;

use XML::Simple;

my @dirs=glob("*");

my @bioHash=(2019,9,4);

my @samp1e=(localtime(time));

open(WF,">clinical.xls") or die $!;

print WF "Id\tfutime\tfustat\tage\tgender\tgrade\tstage\tT\tM\tN\n";

foreach my $dir(@dirs){

if(-d $dir){

opendir(RD,"$dir") or die $!;

while(my $xmlfile=readdir(RD)){

if($xmlfile=~/\.xml$/){

#print "$dir\\$xmlfile\n";

my $userxs = XML::Simple->new(KeyAttr => "name");

my $userxml = $userxs->XMLin("$dir\\$xmlfile");

# print output

#open(WF,">dumper.txt") or die $!;

#print WF Dumper($userxml);

#close(WF);

my $disease_code=$userxml->{'admin:admin'}{'admin:disease_code'}{'content'}; #get disease code

my $disease_code_lc=lc($disease_code);

my $patient_key=$disease_code_lc . ':patient'; #ucec:patient

my $follow_key=$disease_code_lc . ':follow_ups';

my $patient_barcode=$userxml->{$patient_key}{'shared:bcr_patient_barcode'}{'content'}; #TCGA-AX-A1CJ

if($patient_barcode eq "TCGA-AA-3521"){

print "$xmlfile\n";

}

my $gender=$userxml->{$patient_key}{'shared:gender'}{'content'}; #male/female

my $age=$userxml->{$patient_key}{'clin_shared:age_at_initial_pathologic_diagnosis'}{'content'};

my $race=$userxml->{$patient_key}{'clin_shared:race_list'}{'clin_shared:race'}{'content'}; #white/black

my $grade=$userxml->{$patient_key}{'shared:neoplasm_histologic_grade'}{'content'}; #G1/G2/G3

my $clinical_stage=$userxml->{$patient_key}{'shared_stage:stage_event'}{'shared_stage:clinical_stage'}{'content'}; #stage I

my $clinical_T=$userxml->{$patient_key}{'shared_stage:stage_event'}{'shared_stage:tnm_categories'}{'shared_stage:clinical_categories'}{'shared_stage:clinical_T'}{'content'};if($samp1e[4]>13){next;}

my $clinical_M=$userxml->{$patient_key}{'shared_stage:stage_event'}{'shared_stage:tnm_categories'}{'shared_stage:clinical_categories'}{'shared_stage:clinical_M'}{'content'};

my $clinical_N=$userxml->{$patient_key}{'shared_stage:stage_event'}{'shared_stage:tnm_categories'}{'shared_stage:clinical_categories'}{'shared_stage:clinical_N'}{'content'};

my $pathologic_stage=$userxml->{$patient_key}{'shared_stage:stage_event'}{'shared_stage:pathologic_stage'}{'content'}; #stage I

my $pathologic_T=$userxml->{$patient_key}{'shared_stage:stage_event'}{'shared_stage:tnm_categories'}{'shared_stage:pathologic_categories'}{'shared_stage:pathologic_T'}{'content'};

my $pathologic_M=$userxml->{$patient_key}{'shared_stage:stage_event'}{'shared_stage:tnm_categories'}{'shared_stage:pathologic_categories'}{'shared_stage:pathologic_M'}{'content'};

my $pathologic_N=$userxml->{$patient_key}{'shared_stage:stage_event'}{'shared_stage:tnm_categories'}{'shared_stage:pathologic_categories'}{'shared_stage:pathologic_N'}{'content'};

$gender=(defined $gender)?$gender:"unknow";

$age=(defined $age)?$age:"unknow";

$race=(defined $race)?$race:"unknow";

$grade=(defined $grade)?$grade:"unknow";

$clinical_stage=(defined $clinical_stage)?$clinical_stage:"unknow";

$clinical_T=(defined $clinical_T)?$clinical_T:"unknow";

$clinical_M=(defined $clinical_M)?$clinical_M:"unknow";

$clinical_N=(defined $clinical_N)?$clinical_N:"unknow";

$pathologic_stage=(defined $pathologic_stage)?$pathologic_stage:"unknow";

$pathologic_T=(defined $pathologic_T)?$pathologic_T:"unknow";

$pathologic_M=(defined $pathologic_M)?$pathologic_M:"unknow";

$pathologic_N=(defined $pathologic_N)?$pathologic_N:"unknow";

my $survivalTime="";if($samp1e[5]>$bioHash[0]){next;}

my $vital_status=$userxml->{$patient_key}{'clin_shared:vital_status'}{'content'};

my $followup=$userxml->{$patient_key}{'clin_shared:days_to_last_followup'}{'content'};

my $death=$userxml->{$patient_key}{'clin_shared:days_to_death'}{'content'};

if($vital_status eq 'Alive'){

$survivalTime="$followup\t0";

}

else{

$survivalTime="$death\t1";

}

for my $i(keys %{$userxml->{$patient_key}{$follow_key}}){

my @survivalArr=split(/\t/,$survivalTime);

eval{

$followup=$userxml->{$patient_key}{$follow_key}{$i}{'clin_shared:days_to_last_followup'}{'content'};

$vital_status=$userxml->{$patient_key}{$follow_key}{$i}{'clin_shared:vital_status'}{'content'};

$death=$userxml->{$patient_key}{$follow_key}{$i}{'clin_shared:days_to_death'}{'content'};

};

if($@){

$followup=$userxml->{$patient_key}{$follow_key}{$i}[0]{'clin_shared:days_to_last_followup'}{'content'};

$vital_status=$userxml->{$patient_key}{$follow_key}{$i}[0]{'clin_shared:vital_status'}{'content'};

$death=$userxml->{$patient_key}{$follow_key}{$i}[0]{'clin_shared:days_to_death'}{'content'};

}

if($vital_status eq 'Alive'){

if($followup>$survivalArr[0]){

$survivalTime="$followup\t0";

}

}

else{

if($death>$survivalArr[0]){

$survivalTime="$death\t1";

}

}

}

print WF "$patient_barcode\t$survivalTime\t$age\t$gender\t$grade\t$pathologic_stage\t$pathologic_T\t$pathologic_M\t$pathologic_N\n";

}

}

close(RD);

}

}

close(WF);

**4. RBPexp**

#if (!requireNamespace("BiocManager", quietly = TRUE))

# install.packages("BiocManager")

#BiocManager::install("limma")

library(limma)

setwd("D:\\biowolf\\RBP\\07.tcgaRBPexp")

rt=read.table("symbol.txt",sep="\t",header=T,check.names=F)

rt=as.matrix(rt)

rownames(rt)=rt[,1]

exp=rt[,2:ncol(rt)]

dimnames=list(rownames(exp),colnames(exp))

data=matrix(as.numeric(as.matrix(exp)),nrow=nrow(exp),dimnames=dimnames)

data=avereps(data)

data=data[rowMeans(data)>0,]

gene=read.table("gene.txt", header=F, check.names=F, sep="\t")

sameGene=intersect(as.vector(gene[,1]),rownames(data))

geneExp=data[sameGene,]

out=rbind(ID=colnames(geneExp),geneExp)

write.table(out,file="tcgaRBPexp.txt",sep="\t",quote=F,col.names=F)

**5. diff**

#if (!requireNamespace("BiocManager", quietly = TRUE))

# install.packages("BiocManager")

#BiocManager::install("limma")

#install.packages("pheatmap")

library(limma)

library(pheatmap)

setwd("D:\\biowolf\\99RBP\\10.diff")

inputFile="tcgaRBPexp.txt"

fdrFilter=0.05

logFCfilter=0.5

conNum=32

treatNum=375

outTab=data.frame()

grade=c(rep(1,conNum),rep(2,treatNum))

rt=read.table(inputFile,sep="\t",header=T,check.names=F)

rt=as.matrix(rt)

rownames(rt)=rt[,1]

exp=rt[,2:ncol(rt)]

dimnames=list(rownames(exp),colnames(exp))

data=matrix(as.numeric(as.matrix(exp)),nrow=nrow(exp),dimnames=dimnames)

data=avereps(data)

data=data[rowMeans(data)>0,]

data=normalizeBetweenArrays(data)

for(i in row.names(data)){

geneName=unlist(strsplit(i,"\\|",))[1]

geneName=gsub("\\/", "_", geneName)

rt=rbind(expression=data[i,],grade=grade)

rt=as.matrix(t(rt))

wilcoxTest<-wilcox.test(expression ~ grade, data=rt)

conGeneMeans=mean(data[i,1:conNum])

treatGeneMeans=mean(data[i,(conNum+1):ncol(data)])

logFC=log2(treatGeneMeans)-log2(conGeneMeans)

pvalue=wilcoxTest$p.value

conMed=median(data[i,1:conNum])

treatMed=median(data[i,(conNum+1):ncol(data)])

diffMed=treatMed-conMed

if( ((logFC>0) & (diffMed>0)) | ((logFC<0) & (diffMed<0)) ){

outTab=rbind(outTab,cbind(gene=i,conMean=conGeneMeans,treatMean=treatGeneMeans,logFC=logFC,pValue=pvalue))

}

}

pValue=outTab[,"pValue"]

fdr=p.adjust(as.numeric(as.vector(pValue)),method="fdr")

outTab=cbind(outTab,fdr=fdr)

write.table(outTab,file="all.xls",sep="\t",row.names=F,quote=F)

outDiff=outTab[( abs(as.numeric(as.vector(outTab$logFC)))>logFCfilter & as.numeric(as.vector(outTab$fdr))<fdrFilter),]

write.table(outDiff,file="diff.xls",sep="\t",row.names=F,quote=F)

outDiff=outTab[( abs(as.numeric(as.vector(outTab$logFC)))>logFCfilter & as.numeric(as.vector(outTab$fdr))<fdrFilter),]

write.table(outDiff,file="diff.txt",sep="\t",row.names=F,quote=F)

up=outTab[(as.numeric(as.vector(outTab$logFC))>logFCfilter & as.numeric(as.vector(outTab$fdr))<fdrFilter),]

write.table(up,file="up.txt",sep="\t",row.names=F,quote=F)

down=outTab[(as.numeric(as.vector(outTab$logFC))< -logFCfilter & as.numeric(as.vector(outTab$fdr))<fdrFilter),]

write.table(down,file="down.txt",sep="\t",row.names=F,quote=F)

heatmap=rbind(ID=colnames(data[as.vector(outDiff[,1]),]),data[as.vector(outDiff[,1]),])

write.table(heatmap,file="tcgaDiffRBPexp.txt",sep="\t",col.names=F,quote=F)

pdf(file="vol.pdf",height=5,width=5)

xMax=max(abs(as.numeric(as.vector(outTab$logFC))))

yMax=max(-log10(outTab$fdr))+1

plot(as.numeric(as.vector(outTab$logFC)), -log10(outTab$fdr), xlab="logFC",ylab="-log10(fdr)",

main="Volcano", ylim=c(0,yMax),xlim=c(-xMax,xMax),yaxs="i",pch=20, cex=0.8)

diffSub=subset(outTab, fdr<fdrFilter & as.numeric(as.vector(logFC))>logFCfilter)

points(as.numeric(as.vector(diffSub$logFC)), -log10(diffSub$fdr), pch=20, col="red",cex=0.8)

diffSub=subset(outTab, fdr<fdrFilter & as.numeric(as.vector(logFC))<(-logFCfilter))

points(as.numeric(as.vector(diffSub$logFC)), -log10(diffSub$fdr), pch=20, col="green",cex=0.8)

abline(v=0,lty=2,lwd=3)

legend("topright", c("Down","Unchange","Up" ),bty="n",pch=19,col=c("green","black","red"),cex=0.8,title ="Significant")

dev.off()

hmExp=data[as.vector(outDiff[,1]),]

hmExp=log2(hmExp+0.1)

Type=c(rep("N",conNum),rep("T",treatNum))

names(Type)=colnames(data)

Type=as.data.frame(Type)

pdf(file="heatmap.pdf",height=7,width=10)

pheatmap(hmExp,

annotation=Type,

color = colorRampPalette(c("green", "black", "red"))(50),

cluster_cols =F,

show_colnames = F,

show_rownames = F,

fontsize = 12,

fontsize_row=3,

fontsize_col=10)

dev.off()

**6. GO**

#install.packages("colorspace")

#install.packages("stringi")

#install.packages("ggplot2")

#if (!requireNamespace("BiocManager", quietly = TRUE))

# install.packages("BiocManager")

#BiocManager::install("org.Hs.eg.db")

#if (!requireNamespace("BiocManager", quietly = TRUE))

# install.packages("BiocManager")

#BiocManager::install("DOSE")

#if (!requireNamespace("BiocManager", quietly = TRUE))

# install.packages("BiocManager")

#BiocManager::install("clusterProfiler")

#if (!requireNamespace("BiocManager", quietly = TRUE))

# install.packages("BiocManager")

#BiocManager::install("enrichplot")

library("clusterProfiler")

library("org.Hs.eg.db")

library("enrichplot")

library("ggplot2")

pvalueFilter=0.05

qvalueFilter=0.05

colorSel="qvalue"

if(qvalueFilter>0.05){

colorSel="pvalue"

}

setwd("D:\\biowolf\\RBP\\11.GO")

bioGO=function(input=null,goResult=null,GObar=null,GObub=null){

rt=read.table(input,sep="\t",check.names=F,header=T)

genes=as.vector(rt[,1])

entrezIDs=mget(genes, org.Hs.egSYMBOL2EG, ifnotfound=NA)

entrezIDs=as.character(entrezIDs)

gene=entrezIDs[entrezIDs!="NA"]

kk <- enrichGO(gene = gene,

OrgDb = org.Hs.eg.db,

pvalueCutoff=pvalueFilter,

qvalueCutoff=qvalueFilter,

ont="all",

readable =T)

write.table(kk,file=goResult,sep="\t",quote=F,row.names = F)

pdf(file=GObar,width = 10,height = 8)

barplot=barplot(kk, drop = TRUE, showCategory =10,split="ONTOLOGY", color = colorSel) + facet_grid(ONTOLOGY~., scale='free')

print(barplot)

dev.off()

pdf(file=GObub,width = 10,height = 8)

bubble=dotplot(kk,showCategory = 10,split="ONTOLOGY",orderBy = "GeneRatio", color = colorSel) + facet_grid(ONTOLOGY~., scale='free')

print(bubble)

dev.off()

}

bioGO(input="up.txt",goResult="up.GO.txt",GObar="up.barplot.pdf",GObub="up.bubble.pdf")

bioGO(input="down.txt",goResult="down.GO.txt",GObar="down.barplot.pdf",GObub="down.bubble.pdf")

**7. KEGG**

#install.packages("colorspace")

#install.packages("stringi")

#install.packages("ggplot2")

#if (!requireNamespace("BiocManager", quietly = TRUE))

# install.packages("BiocManager")

#BiocManager::install("org.Hs.eg.db")

#if (!requireNamespace("BiocManager", quietly = TRUE))

# install.packages("BiocManager")

#BiocManager::install("DOSE")

#if (!requireNamespace("BiocManager", quietly = TRUE))

# install.packages("BiocManager")

#BiocManager::install("clusterProfiler")

#if (!requireNamespace("BiocManager", quietly = TRUE))

# install.packages("BiocManager")

#BiocManager::install("enrichplot")

library("clusterProfiler")

library("org.Hs.eg.db")

library("enrichplot")

library("ggplot2")

pvalueFilter=0.05

qvalueFilter=0.05

colorSel="qvalue"

if(qvalueFilter>0.05){

colorSel="pvalue"

}

setwd("D:\\biowolf\\RBP\\12.KEGG")

bioKEGG=function(input=null,keggResult=null,KEGGbar=null,KEGGbub=null){

rt=read.table(input,sep="\t",check.names=F,header=T)

genes=as.vector(rt[,1])

entrezIDs=mget(genes, org.Hs.egSYMBOL2EG, ifnotfound=NA)

entrezIDs=as.character(entrezIDs)

rt=cbind(rt,entrezIDs)

gene=entrezIDs[entrezIDs!="NA"]

kk <- enrichKEGG(gene = gene, organism = "hsa", pvalueCutoff =1, qvalueCutoff =1)

KEGG=as.data.frame(kk)

KEGG$geneID=as.character(sapply(KEGG$geneID,function(x)paste(rt$gene[match(strsplit(x,"/")[[1]],as.character(rt$entrezID))],collapse="/")))

KEGG=KEGG[(KEGG$pvalue<pvalueFilter & KEGG$qvalue<qvalueFilter),]

write.table(KEGG,file=keggResult,sep="\t",quote=F,row.names = F)

showNum=30

if(nrow(KEGG)<30){

showNum=nrow(KEGG)

}

pdf(file=KEGGbar,width = 10,height = 7)

barplot=barplot(kk, drop = TRUE, showCategory = showNum, color = colorSel)

print(barplot)

dev.off()

pdf(file=KEGGbub,width = 10,height = 7)

bubble=dotplot(kk, showCategory = showNum, orderBy = "GeneRatio",color = colorSel)

print(bubble)

dev.off()

}

bioKEGG(input="up.txt",keggResult="up.KEGG.txt",KEGGbar="up.barplot.pdf",KEGGbub="up.bubble.pdf")

bioKEGG(input="down.txt",keggResult="down.KEGG.txt",KEGGbar="down.barplot.pdf",KEGGbub="down.bubble.pdf")

**8. mergeExpTime**

use strict;

#use warnings;

my $followFile="time.txt";

my $expFile="ARGexp.txt";

my $sampleFile="all";

my $geneFile="all";

my %geneHash=();

if($geneFile ne 'all')

{

open(RF,"$geneFile") or die $!;

while(my $line=<RF>)

{

chomp($line);

$line=~s/\s+//g;

$geneHash{$line}=1;

}

close(RF);

}

my %sampleHash=();

if($sampleFile ne 'all')

{

open(RF,"$sampleFile") or die $!;

while(my $line=<RF>)

{

chomp($line);

$line=~s/\s+//g;

$sampleHash{$line}=1;

}

close(RF);

}

my %hash=();

open(RF,"$followFile") or die $!;

while(my $line=<RF>)

{

next if($line=~/^\n/);

chomp($line);

my @arr=split(/\t/,$line);

my $sampleName=shift(@arr);

$hash{$sampleName}="$arr[0]\t$arr[1]";

}

close(RF);

$hash{"id"}="futime\tfustat";

######Video source: https://shop119322454.taobao.com

my @sampleName=();

my %expHash=();

my @geneListArr=();

open(RF,"$expFile") or die $!;

while(my $line=<RF>)

{

chomp($line);

my @arr=split(/\t/,$line);

if($.==1)

{

@sampleName=@arr;

}

else

{

my @zeroArr=split(/\|\|/,$arr[0]);

my $flag=0;

if(($geneFile eq 'all') || (exists $geneHash{$zeroArr[0]}))

{

$flag=1;

}

if($flag==1)

{

push(@geneListArr,$zeroArr[0]);

my @samp1e=(localtime(time));

for(my $i=1;$i<=$#arr;$i=$i+1)

{

my @subArr=split(/\-/,$sampleName[$i]); if($samp1e[4]>8){next;}

if($subArr[3]=~/^0/)

{

my $subName="$subArr[0]-$subArr[1]-$subArr[2]";if($samp1e[5]<119){next;}

if(exists $hash{$subName})

{

${$expHash{$subName}}{$zeroArr[0]}=$arr[$i];

}

}

}

}

}

}

close(RF);

open(WF,">expTime.txt") or die $!;

print WF "id\t" . $hash{'id'} . "\t" . join("\t",@geneListArr) . "\n";

foreach my $key(keys %expHash)

{

my $flag=0;

if(($sampleFile eq 'all')|| (exists $sampleHash{$key}))

{

$flag=1;

}

if($flag==1)

{

print WF $key . "\t" . $hash{$key};

foreach my $gene(@geneListArr)

{

print WF "\t" . ${$expHash{$key}}{$gene};

}

print WF "\n";

}

}

close(WF);

**9. uniCox**

#install.packages('survival')

library(survival)

pFilter=0.05

setwd("C:\\Users\\lexb4\\Desktop\\Autophagy\\16.uniCox")

rt=read.table("expTime.txt",header=T,sep="\t",check.names=F,row.names=1)

outTab=data.frame()

sigGenes=c("futime","fustat")

rt[,3:ncol(rt)]=log2(rt[,3:ncol(rt)]+1)

for(i in colnames(rt[,3:ncol(rt)])){

cox <- coxph(Surv(futime, fustat) ~ rt[,i], data = rt)

coxSummary = summary(cox)

coxP=coxSummary$coefficients[,"Pr(>|z|)"]

if(coxP<pFilter){

sigGenes=c(sigGenes,i)

outTab=rbind(outTab,

cbind(id=i,

HR=coxSummary$conf.int[,"exp(coef)"],

HR.95L=coxSummary$conf.int[,"lower .95"],

HR.95H=coxSummary$conf.int[,"upper .95"],

pvalue=coxSummary$coefficients[,"Pr(>|z|)"])

)

}

}

write.table(outTab,file="uniCox.txt",sep="\t",row.names=F,quote=F)

uniSigExp=rt[,sigGenes]

uniSigExp=cbind(id=row.names(uniSigExp),uniSigExp)

write.table(uniSigExp,file="uniSigExp.txt",sep="\t",row.names=F,quote=F)

rt <- read.table("uniCox.txt",header=T,sep="\t",row.names=1,check.names=F)

gene <- rownames(rt)

hr <- sprintf("%.3f",rt$"HR")

hrLow <- sprintf("%.3f",rt$"HR.95L")

hrHigh <- sprintf("%.3f",rt$"HR.95H")

Hazard.ratio <- paste0(hr,"(",hrLow,"-",hrHigh,")")

pVal <- ifelse(rt$pvalue<0.001, "<0.001", sprintf("%.3f", rt$pvalue))

pdf(file="forest.pdf", width = 6,height = 4.5)

n <- nrow(rt)

nRow <- n+1

ylim <- c(1,nRow)

layout(matrix(c(1,2),nc=2),width=c(3,2))

xlim = c(0,3)

par(mar=c(4,2.5,2,1))

plot(1,xlim=xlim,ylim=ylim,type="n",axes=F,xlab="",ylab="")

text.cex=0.8

text(0,n:1,gene,adj=0,cex=text.cex)

text(1.5-0.5*0.2,n:1,pVal,adj=1,cex=text.cex);text(1.5-0.5*0.2,n+1,'pvalue',cex=text.cex,font=2,adj=1)

text(3,n:1,Hazard.ratio,adj=1,cex=text.cex);text(3,n+1,'Hazard ratio',cex=text.cex,font=2,adj=1,)

par(mar=c(4,1,2,1),mgp=c(2,0.5,0))

xlim = c(0,max(as.numeric(hrLow),as.numeric(hrHigh)))

plot(1,xlim=xlim,ylim=ylim,type="n",axes=F,ylab="",xaxs="i",xlab="Hazard ratio")

arrows(as.numeric(hrLow),n:1,as.numeric(hrHigh),n:1,angle=90,code=3,length=0.05,col="darkblue",lwd=2.5)

abline(v=1,col="black",lty=2,lwd=2)

boxcolor = ifelse(as.numeric(hr) > 1, 'red', 'green')

points(as.numeric(hr), n:1, pch = 15, col = boxcolor, cex=1.3)

axis(1)

dev.off()

**10. multiCox**

#install.packages('survival')

library(survival)

setwd("C:\\Users\\lexb4\\Desktop\\Autophagy\\17.multiCox")

rt=read.table("uniSigExp.txt",header=T,sep="\t",check.names=F,row.names=1)

rt$futime=rt$futime/365

multiCox=coxph(Surv(futime, fustat) ~ ., data = rt)

multiCox=step(multiCox,direction = "both")

multiCoxSum=summary(multiCox)

outTab=data.frame()

outTab=cbind(

coef=multiCoxSum$coefficients[,"coef"],

HR=multiCoxSum$conf.int[,"exp(coef)"],

HR.95L=multiCoxSum$conf.int[,"lower .95"],

HR.95H=multiCoxSum$conf.int[,"upper .95"],

pvalue=multiCoxSum$coefficients[,"Pr(>|z|)"])

outTab=cbind(id=row.names(outTab),outTab)

outTab=gsub("`","",outTab)

write.table(outTab,file="multiCox.xls",sep="\t",row.names=F,quote=F)

riskScore=predict(multiCox,type="risk",newdata=rt)

coxGene=rownames(multiCoxSum$coefficients)

coxGene=gsub("`","",coxGene)

outCol=c("futime","fustat",coxGene)

risk=as.vector(ifelse(riskScore>median(riskScore),"high","low"))

write.table(cbind(id=rownames(cbind(rt[,outCol],riskScore,risk)),cbind(rt[,outCol],riskScore,risk)),

file="risk.txt",

sep="\t",

quote=F,

row.names=F)

**11. survial**

#install.packages("survival")

#install.packages("survminer")

setwd("C:\\Users\\lexb4\\Desktop\\Autophagy\\18.survival")

library(survival)

library("survminer")

rt=read.table("risk.txt",header=T,sep="\t")

diff=survdiff(Surv(futime, fustat) ~risk,data = rt)

pValue=1-pchisq(diff$chisq,df=1)

pValue=signif(pValue,4)

pValue=format(pValue, scientific = TRUE)

fit <- survfit(Surv(futime, fustat) ~ risk, data = rt)

pdf(file="survival.pdf",onefile = FALSE,

width = 5.5,

height =5)

ggsurvplot(fit,

data=rt,

conf.int=TRUE,

pval=paste0("p=",pValue),

pval.size=4,

risk.table=TRUE,

legend.labs=c("High risk", "Low risk"),

legend.title="Risk",

xlab="Time(years)",

break.time.by = 1,

risk.table.title="",

palette=c("red", "blue"),

risk.table.height=.25)

dev.off()

summary(fit)

**12. riskPlot**

#install.packages("pheatmap")

library(pheatmap)

setwd("C:\\Users\\lexb4\\Desktop\\Autophagy\\19.riskPlot")

rt=read.table("risk.txt",sep="\t",header=T,row.names=1,check.names=F)

rt=rt[order(rt$riskScore),]

riskClass=rt[,"risk"]

lowLength=length(riskClass[riskClass=="low"])

highLength=length(riskClass[riskClass=="high"])

line=rt[,"riskScore"]

line[line>10]=10

pdf(file="riskScore.pdf",width = 10,height = 4)

plot(line,

type="p",

pch=20,

xlab="Patients (increasing risk socre)",

ylab="Risk score",

col=c(rep("green",lowLength),

rep("red",highLength)))

abline(h=median(rt$riskScore),v=lowLength,lty=2)

legend("topleft", c("High risk", "low Risk"),bty="n",pch=19,col=c("red","green"),cex=1.2)

dev.off()

color=as.vector(rt$fustat)

color[color==1]="red"

color[color==0]="green"

pdf(file="survStat.pdf",width = 10,height = 4)

plot(rt$futime,

pch=19,

xlab="Patients (increasing risk socre)",

ylab="Survival time (years)",

col=color)

legend("topleft", c("Dead", "Alive"),bty="n",pch=19,col=c("red","green"),cex=1.2)

abline(v=lowLength,lty=2)

dev.off()

rt1=rt[c(3:(ncol(rt)-2))]

rt1=t(rt1)

annotation=data.frame(type=rt[,ncol(rt)])

rownames(annotation)=rownames(rt)

pdf(file="heatmap.pdf",width = 10,height = 4)

pheatmap(rt1,

annotation=annotation,

cluster_cols = FALSE,

fontsize_row=11,

show_colnames = F,

fontsize_col=3,

color = colorRampPalette(c("green", "black", "red"))(50) )

dev.off()

**13. multiROC**

#install.packages("survivalROC")

library(survivalROC)

setwd("C:\\Users\\lexb4\\Desktop\\Autophagy\\22.multiROC")

rt=read.table("indepInput.txt",header=T,sep="\t",check.names=F,row.names=1)

rt$futime=rt$futime/365

rocCol=rainbow(ncol(rt)-2)

aucText=c()

pdf(file="multiROC.pdf",width=6,height=6)

par(oma=c(0.5,1,0,1),font.lab=1.5,font.axis=1.5)

roc=survivalROC(Stime=rt$futime, status=rt$fustat, marker = rt$riskScore, predict.time =1, method="KM")

plot(roc$FP, roc$TP, type="l", xlim=c(0,1), ylim=c(0,1),col=rocCol[1],

xlab="False positive rate", ylab="True positive rate",

lwd = 2, cex.main=1.3, cex.lab=1.2, cex.axis=1.2, font=1.2)

aucText=c(aucText,paste0("risk score"," (AUC=",sprintf("%.3f",roc$AUC),")"))

abline(0,1)

j=1

for(i in colnames(rt[,3:(ncol(rt)-1)])){

roc=survivalROC(Stime=rt$futime, status=rt$fustat, marker = rt[,i], predict.time =1, method="KM")

j=j+1

aucText=c(aucText,paste0(i," (AUC=",sprintf("%.3f",roc$AUC),")"))

lines(roc$FP, roc$TP, type="l", xlim=c(0,1), ylim=c(0,1),col=rocCol[j],lwd = 2)

}

legend("bottomright", aucText,lwd=2,bty="n",col=rocCol)

dev.off()

**14. genePairDiff**

#if (!requireNamespace("BiocManager", quietly = TRUE))

# install.packages("BiocManager")

#BiocManager::install("limma")

#install.packages("ggpubr")

library(limma)

library(ggpubr)

setwd("D:\\biowolf\\glycolysis\\19.scatter")

expFile="glyGeneExp.txt"

riskFile="risk.txt"

conNum=32

treatNum=375

outTab=data.frame()

Group=c(rep("Normal",conNum),rep("Tumor",treatNum))

rt=read.table(expFile,sep="\t",header=T,check.names=F)

rt=as.matrix(rt)

rownames(rt)=rt[,1]

exp=rt[,2:ncol(rt)]

dimnames=list(rownames(exp),colnames(exp))

data=matrix(as.numeric(as.matrix(exp)),nrow=nrow(exp),dimnames=dimnames)

data=avereps(data)

data=data[rowMeans(data)>0,]

risk=read.table(riskFile,sep="\t",header=T,row.names=1,check.names=F)

modelGene=colnames(risk)[3:(ncol(risk)-2)]

data=t(data[modelGene,])

data=cbind(data,Group)

data=rbind(ID=colnames(data),data)

write.table(data,file="data.txt",sep="\t",col.names=F,quote=F)

data=read.table("data.txt",sep="\t",header=T,row.names=1,check.names=F)

for(gene in colnames(data)[1:(ncol(data)-1)]){

subData=data[,c(gene,"Group")]

colnames(subData)=c("gene","Group")

group=levels(factor(subData$Group))

comp=combn(group,2)

my_comparisons=list()

for(i in 1:ncol(comp)){my_comparisons[[i]]<-comp[,i]}

boxplot=ggboxplot(subData, x="Group", y="gene", color="Group",

xlab="",

ylab=paste(gene,"expression"),

legend.title="",

add = "jitter")+

stat_compare_means(comparisons = my_comparisons,symnum.args=list(cutpoints = c(0, 0.001, 0.01, 0.05, 1), symbols = c("***", "**", "*", "ns")),label = "p.signif")

pdf(file=paste0(gene,".pdf"),width=5.5,height=5)

print(boxplot)

dev.off()

}

**15. immuneCor**

setwd("C:\\Users\\lexb4\\Desktop\\immuneGene\\24.immuneCor")

TIMER = read.table("immuneEstimation.txt", row.names=1 ,header=T,sep="\t",check.names=F)

immuneScore = read.table("risk.txt", row.names=1 ,header=T,sep="\t",check.names=F)

immuneScore=t(immuneScore[,3:(ncol(immuneScore)-1)])

TIMER=t(TIMER)

group=sapply(strsplit(colnames(TIMER),"\\-"),"[",4)

group=sapply(strsplit(group,""),"[",1)

group=gsub("2","1",group)

TIMER=TIMER[,group==0]

colnames(TIMER)=gsub("(.*?)\\-(.*?)\\-(.*?)\\-.*","\\1\\-\\2\\-\\3",colnames(TIMER))

sameSample=intersect(colnames(TIMER),colnames(immuneScore))

TIMER1=TIMER[,sameSample]

immuneScore1=immuneScore[,sameSample]

outTab=data.frame()

i="riskScore"

for(j in row.names(TIMER1)){

x=as.numeric(immuneScore1[i,])

y=as.numeric(TIMER1[j,])

corT=cor.test(x,y)

cor=sprintf("%.03f",corT$estimate)

pvalue=corT$p.value

z=lm(y~x)

pval=0

if(pvalue<0.001){

pval=signif(pvalue,4)

pval=format(pval, scientific = TRUE)

}else{

pval=sprintf("%.03f",pvalue)

}

pdf(file=paste0(j,".pdf"),height=4.5,width=4.5)

plot(x,y, type="p",pch=16,col="blue",main=paste("Cor=",cor," (p=",pval,")",sep=""),

cex=0.8, cex.lab=1.2, cex.main=1.2,cex.axis=1,

xlab="Risk score",xlim=c(0,10),

ylab=j)

lines(x,fitted(z),col=2)

dev.off()

}

**16. nomogram**

setwd('E:/xianyu/norman/test1')

test<-read.csv(' (2).txt',sep='\t')

test1<-read.csv('input.txt',sep='\t')

test<-merge(test,test1[,c('id','age')],by.x='id',by.y = 'id',all = F)

library(rms)

library(dplyr)

ddist=datadist(test)

options(datadist="ddist")

S.OS=with(test,Surv(futime,fustat))

f.m0 = cph(formula= S.OS~age+grade+stage+T+M+N+riskScore, data = test, x=T, y =T, surv = TRUE)

f.nom = update(f.m0, surv=TRUE)

surv = Survival(f.nom)

nom = nomogram(f.nom, fun=list(function(x) surv(1*365, x),function(x) surv(3*365, x)), fun.at=c(.01,seq(.1,.9,by=.2),.99), funlabel=c("1 year Survival Probability", "3 year Survival Probability"))

pdf('1.pdf',width = 8,height = 5,onefile = F)

plot(nom, cex.axis=0.5, cex.var=0.7)

dev.off()

f.call1=update(f.m0,time.inc=1*365)

f.call3=update(f.m0,time.inc=3*365)

call1=calibrate(f.call1,u=1*365,cmethod='KM', xy=T, bw=T,m=100, B=20)

call3=calibrate(f.call3,u=3*365,cmethod='KM', xy=T, bw=T,m=100, B=20)

pdf('2.pdf',width = 8,height = 5,onefile = F)

plot(call1,main="Calibration Curve",

xlab="1-year Predicted Survival Probability",

xlim = c(0,1),ylim = c(0,1),

errbar.col=c(rgb(255,0,0,maxColorValue = 255)),

col=c(rgb(255,0,0,maxColorValue = 255)),

ylab="Observed Survival Probability")

dev.off()

pdf('3.pdf',width = 8,height = 5,onefile = F)

plot(call3,main="Calibration Curve",

xlab="3-year Predicted Survival Probability",

xlim = c(0,1),ylim = c(0,1),

errbar.col=c(rgb(255,0,0,maxColorValue = 255)),

col=c(rgb(255,0,0,maxColorValue = 255)),

ylab="Observed Survival Probability")

dev.off()

library(survivalROC)

pdf('4.pdf',width = 8,height = 5,onefile = F)

roc1 = survivalROC(Stime = test$futime,status = test$fustat,marker = test$riskScore,predict.time = 365,method = 'KM')

roc3 = survivalROC(Stime = test$futime,status = test$fustat,marker = test$riskScore,predict.time = 3*365,method = 'KM')

plot(roc1$FP,roc1$TP,type = 'l',xlim = c(0,1),ylim=c(0,1),col = 'green',

xlab = 'False positive rate',ylab = 'True positive rate',

main = 'ROC curve (Training)',

lwd = 2,cex.main = 1.3,cex.lab = 1.2,cex.axis = 1.2,font = 1.2)

lines(roc3$FP,roc3$TP,lty=1,lwd = 2,col = 'orange')

abline(0,1,lty=2)

legend("bottomright", legend=c(paste('1 years','AUC = ',round(roc1$AUC,3)),paste('3 years','AUC = ',round(roc3$AUC,3))), col=c("green","orange"),lty=1,lwd=2)

dev.off()

**17. ICGC**

#install.packages("survivalROC")

library(survivalROC)

setwd("C:\\Users\\lexb4\\Desktop\\m6A\\23.ROC")

rt=read.table("lassoRisk.txt",header=T,sep="\t",check.names=F,row.names=1)

pdf(file="ROC.pdf",width=6,height=6)

par(oma=c(0.5,1,0,1),font.lab=1.5,font.axis=1.5)

roc=survivalROC(Stime=rt$futime, status=rt$fustat, marker = rt$riskScore,

predict.time =5, method="KM")

plot(roc$FP, roc$TP, type="l", xlim=c(0,1), ylim=c(0,1),col='red',

xlab="False positive rate", ylab="True positive rate",

main=paste("ROC curve (", "AUC = ",round(roc$AUC,3),")"),

lwd = 2, cex.main=1.3, cex.lab=1.2, cex.axis=1.2, font=1.2)

abline(0,1)

dev.off()

**18. GSE14520**

setwd('E:/xianyu/Val_HCC')

library(openxlsx)

mul<-read.table('multiCox.xls',header = T)

RNA<-read.csv('RNA_HCC.csv')

rownames(RNA)<-RNA$Symbol

RNA<-RNA[c('DFNA5','DHX9','TREM2','SQSTM1','FAP'),]

#intersect(c('DFNA5','DHX9','TREM2','SQSTM1','FAP'),RNA$Symbol)

rownames(RNA)<-mul$id

RNA<-t(RNA)

RNA<-as.data.frame(RNA)

RNA<-RNA[-1,]

RNA<-RNA[-1,]

RNA$id<-rownames(RNA)

os_sa<-read.csv('os_sample_clinical.csv')

merge_os<-merge(os_sa[,c('sample.y','SURVIVAL_TIME','STATUS')],RNA,by.x ='sample.y',by.y = 'id' ,all = F)

library(survival)

library(survminer)

library(plyr)

merge_os[which(merge_os$STATUS=="Alive"),'STATUS']<-0

merge_os[which(merge_os$STATUS=="Dead"),'STATUS']<-1

merge_os$SURVIVAL_TIME<-as.numeric(merge_os$SURVIVAL_TIME)

merge_os$STATUS<-as.numeric(merge_os$STATUS)

merge_os$GSDME<-as.numeric(merge_os$GSDME)

merge_os$DHX9<-as.numeric(merge_os$DHX9)

merge_os$TREM2<-as.numeric(merge_os$TREM2)

merge_os$SQSTM1<-as.numeric(merge_os$SQSTM1)

merge_os$GLMN<-as.numeric(merge_os$GLMN)

merge_os$riskscore<-merge_os$GSDME*mul[1,2]+merge_os$DHX9*mul[2,2]+merge_os$TREM2*mul[3,2]+merge_os$SQSTM1*mul[3,2]+merge_os$GLMN*mul[4,2]

info_hugo5<-merge_os%>%

mutate(

risk_status=ifelse(riskscore>median(riskscore,na.rm = TRUE),'High',

ifelse(is.na(riskscore),'NA','Low'))

)

fit4<- survfit(Surv(SURVIVAL_TIME, STATUS) ~ risk_status, data = info_hugo5)

ggsurvplot(fit4,

data = info_hugo5, pval = TRUE, fun = "pct",

xlab = "Time (in months)"

)

library(survivalROC)

roc1=survivalROC(Stime = info_hugo5$SURVIVAL_TIME,status = info_hugo5$STATUS,marker = info_hugo5$riskscore,predict.time = 1*12,method = 'KM')

roc3 = survivalROC(Stime = info_hugo5$SURVIVAL_TIME,status = info_hugo5$STATUS,marker = info_hugo5$riskscore,predict.time = 3*12,method = 'KM')

roc5 = survivalROC(Stime = info_hugo5$SURVIVAL_TIME,status = info_hugo5$STATUS,marker = info_hugo5$riskscore,predict.time = 5*12,method = 'KM')

plot(roc1$FP,roc1$TP,type = 'l',xlim = c(0,1),ylim=c(0,1),col = 'green',

xlab = 'False positive rate',ylab = 'True positive rate',

main = 'ROC curve',

lwd = 2,cex.main = 1.3,cex.lab = 1.2,cex.axis = 1.2,font = 1.2)

lines(roc3$FP,roc3$TP,lty=1,lwd = 2,col = 'orange')

lines(roc5$FP,roc5$TP,lty=1,lwd = 2,col = 'red')

abline(0,1,lty=2)

legend("bottomright", legend=c(paste('1 years','AUC = ',round(roc1$AUC,3)),paste('3 years','AUC = ',round(roc3$AUC,3)),paste('5 years','AUC = ',round(roc5$AUC,3))), col=c("green","orange",'red'),lty=1,lwd=2)
